# Bovine Serum Albumin-Coated Niclosamide-Zein Nanoparticles as Potential Injectable Medicine against COVID-19

**DOI:** 10.3390/ma14143792

**Published:** 2021-07-07

**Authors:** Sanoj Rejinold N, Goeun Choi, Huiyan Piao, Jin-Ho Choy

**Affiliations:** 1 Intelligent Nanohybrid Materials Laboratory (INML), Institute of Tissue Regeneration Engineering (ITREN), Dankook University, Cheonan 31116, Korea; sanojrejinold@dankook.ac.kr (S.R.N.); goeun.choi@dankook.ac.kr (G.C.); 12192032@dankook.ac.kr (H.P.); 2 College of Science and Technology, Dankook University, Cheonan 31116, Korea; 3 Department of Nanobiomedical Science and BK21 PLUS NBM Global Research Center for Regenerative Medicine, Dankook University, Cheonan 31116, Korea; 4 Department of Pre-medical Course, College of Medicine, Dankook University, Cheonan 31116, Korea; 5 Tokyo Tech World Research Hub Initiative (WRHI), Institute of Innovative Research, Tokyo Institute of Technology, Yokohama 226-8503, Japan

**Keywords:** Zein, niclosamide, albumin, COVID-19, endothelial glycocalyx damage

## Abstract

(1) Background: COVID-19 has affected millions of people worldwide, but countries with high experimental anti-SARS-CoV-2 vaccination rates among the general population respectively show progress in achieving general herd immunity in the population (a combination of natural and vaccine-induced acquired immunity), resulting in a significant reduction in both newly detected infections and mortality rates. However, the longevity of the vaccines’ ability to provide protection against the ongoing pandemic is still unclear. Therefore, it is of utmost importance to have new medications to fight against the pandemic at the earliest point possible. Recently, it has been found that repurposing already existing drugs could, in fact, be an ideal strategy to formulate effective medication for COVID-19. Though there are many FDA-approved drugs, it has been found that niclosamide (NIC), an anthelmintic drug, has significantly high potential against the SARS-CoV-2 virus. (2) Methods: Here we deployed a simple self-assembling technique through which Zein nanoparticles were successfully used to encapsulate NIC, which was then coated with bovine serum albumin (BSA) in order to improve the drugs’ stability, injectablity, and selectivity towards the virus-infected cells. (3) Results: The particle size for the BSA-stabilized Zein-NIC nanohybrid was found to be less than 200 nm, with excellent colloidal stability and sustained drug release properties. In addition, the nanohybrid showed enhanced drug release behavior under serum conditions, indicating that such a hybrid drug delivery system could be highly beneficial for treating COVID-19 patients suffering from high endothelial glycocalyx damage followed by a cytokine storm related to the severe inflammations.

## 1. Introduction

The whole world has been witnessing a devastating pandemic in the form of coronavirus disease 2019 (COVID-19), caused by the SARS-CoV-2 virus [1,2,3,4,5,6,7,8,9,10,11,12], ever since it was first discovered in China’s Wuhan city in 2019 [13,14,15,16,17]. As of 5 July 2021, there were ~184 M individuals worldwide contaminated with this lethal viral disease and 3.98 M people dead. Although vaccinations have been initiated globally, the current situation in many countries where the second and third waves have hit lethally [18] demands an alternative medical strategy that, together with vaccination, can successfully subside the pandemic. However, the longevity of the capacity of vaccination to induce long-term immunity to combat COVID-19 is still unknown.

While keeping this in mind, we should, however, understand that there have been various approaches implemented by the scientists across the world to develop novel formulations that could be useful as antiviral medications to deal with COVID-19. One of the most useful strategies is repurposing [19,20,21] already existing FDA-approved drug candidates involving biomaterials, such as organic or inorganic ones, in order to improve their therapeutic efficacy. However, one has to rationally select highly biocompatible and efficient drug carriers to selectively target the infected cells, and thereby minimize the side effects on the unaffected cells or tissues. 

From among the wide varieties of antiviral drugs that could potentially be used for repurposing towards the treatment of COVID-19, scientists have discovered an anthelmintic niclosamide (NIC) drug that possesses very strong antiviral effects [22] with a minimum therapeutic concentration. Basically, NIC can exert an antiviral mechanism through extra- and intra-cellular pathways. In the extracellular mechanism, NIC can interfere with the human angiotensin converting enzyme-2 (h-ACE2) by which the SARS-CoV-2 virus enters the cells. NIC can block this endocytosis process, and once these NIC molecules have permeated inside the infected cells, they can further alter the autophagy and halt the viral replication, thereby preventing the spread of viral particles within the cells [23]. In addition, NIC can block syncytia by suppressing the activity of transmembrane protein 16F (TMEM16F), also known as anoctamin 6, a calcium-activated ion channel and scramblase that is responsible for phosphatidylserine exposure on the cell surface [24]. 

Although NIC has very high anti-viral efficacy, the major drawback is its extremely poor water solubility, which thereby limits the *in-vivo* efficacy [25]. There have been various methodologies proposed, such as physical and chemical modification, to alter the pharmacokinetics (PK) of NIC, as suggested recently by our research group [23,26]. However, in general, one has to be very careful when selecting an appropriate drug delivery carrier for the repurposing applications. Here, we focused on the development of an injectable anti-COVID formulation in order to facilitate fast delivery into the blood, wherein the damaged endothelial glycocalyx in COVID-19 patients [27,28,29,30,31,32] would preferably enable an enhanced uptake of the NIC drug out of the injected formulation.

**Scheme 1:**
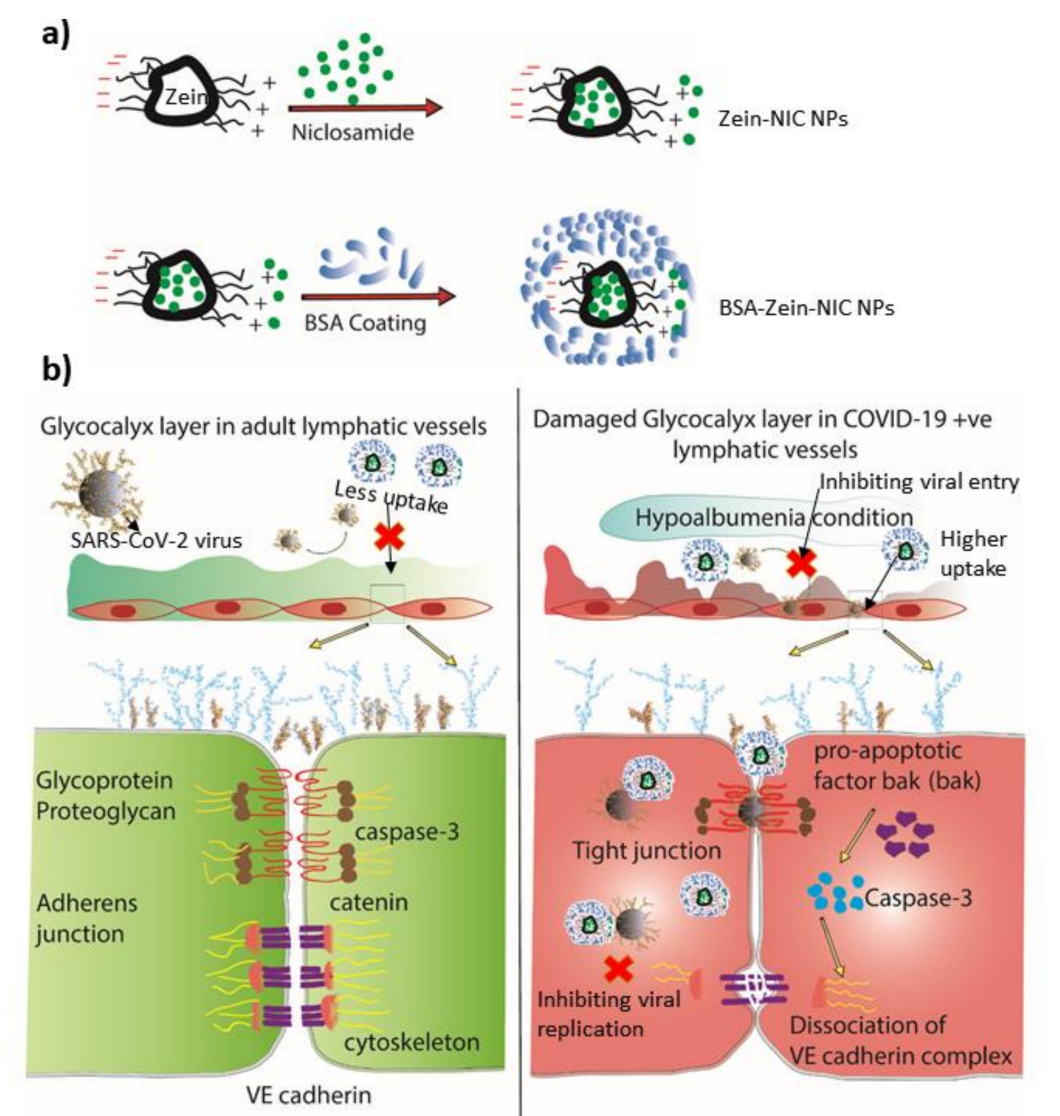
(**a**) Synthetic strategy of injectable NIC formulation with Zein and BSA; (**b**) proposal for how the injectable NIC formulation could be useful for treating COVID-19 patients with a damaged glycocalyx followed by a cytokine storm, especially in severely affected patients.

Though there are different kinds of drug carriers reported for NIC formulations, such as inorganic montmorillonite (MMT), dehydrotalcite (DHT) [23,26], and chitosan [33], here we chose Zein, a biocompatible phytoprotein, mainly because it is highly biocompatible and degradable and it has been extensively studied for various biomedical applications [34,35,36,37,38,39]. In addition, there are several reports suggesting that Zein-based hybrids are well-tolerated for human applications. In order to further improve the selectivity of these hybrid NPs, bovine serum albumin (BSA), a natural biomolecule, was coated to finally make BSA-Zein-NIC NPs (Scheme 1a). It is worth mentioning here that BSA-based nano-biomaterials have been well-studied in the drug delivery field owing to their specificity of being able to get into an infected site.[40] In addition, albumin can easily be taken up by the virus-infected cells since a cytokine storm follows glycocalyx damage in elderly and severely affected COVID-19 patients [28,29,30,31]. In this context, we believed that BSA-coated Zein-NIC NPs would be highly preferred as an ideal injectable formulation for treating COVID-19 patients.

Here, the major research questions we focused on addressing can be stated as follows: (1) How can NIC be repurposed as an injectable formulation with Zein and BSA biomaterials? (2) How do Zein and BSA facilitate sustained NIC release? (3) How can this injectable formulation be useful in treating COVID-19 patients? The research hypotheses were as follows: (1) The NIC molecules in solution, being negatively charged, would undergo electrostatic interaction with the positively charged Zein with a polypeptide structure, forming strong drug-loaded NPs, whereas the BSA would suitable to be coated physically on the surface owing to its anionic surface charge. (2) Since the Zein protein is swellable under an aqueous solution, it was expected that NIC drug molecules immobilized in Zein would be released out in a sustained manner, whereas the coated BSA would enable selective targeting toward viral infected cells. (3) We therefore propose that injectable BSA-Zein-NIC NPs are highly beneficial for COVID-19 patients, mainly due to the fact that albumin uptake is higher in COVID-19 patients, as the endothelial glycocalyx has been reported to be partly or completely perturbed in these patients, as shown in Scheme 1b.

## 2. Materials and Methods

### 2.1. Materials

Niclosamide was delivered from Derivados Quimicos, Murcia, Spain. Ethyl alcohol (99.9%) and isopropyl alcohol (IPA) were bought from Daejung Chemicals (Daejung Chemicals, Gyeonggi-do, Korea). Zein and BSA were purchased from Sigma-Aldrich (Sigma-Aldrich, St. Luis, MO, USA) and TCI (Tokyo Chemical Industries, Tokyo, Japan,) respectively. Phosphate buffered saline tablets were purchased from Gibco (Gibco^TM^, Thermo Fisher Scientific, Paisley, UK).

### 2.2. Preparation of Zein-NIC NPs

Initially, Zein stock solution was produced as follows. Zein (Sigma Aldrich, St. Luis, MO, USA), was dissolved in 70% IPA (Daejung Chemicals, Gyeonggi-do, Korea) with a concentration of 10 mg/mL (Zein stock solution), while NIC was dissolved in IPA with a concentration of 5mg/mL (NIC stock solution), respectively. Then, 1 mL of dissolved Zein/IPA (containing 10 mg Zein) solution was treated with 200 µL of NIC (Derivados Quimicos, Murcia, Spain) in IPA containing 1.25 mg NIC, which was then gently mixed using a vortex mixer (Scientific Industries Inc., Bohemia, NY, USA) for about 1 min. This mixture was directly added into 8.75 mL distilled water, forming an opalescent solution indicative of Zein-NIC NPs. This solution was further evaporated using a rotary evaporator (Heidolph Instruments Gm bH & Co. KG, Schwabach, Germany) for 10–15 min and then freeze-dried at −53 °C using a freeze drier machine (ILSHIN BIOBASE, Gyeonggi-do, Korea) for 2 days in order to obtain the powder form. 

As shown in the supporting information (Figure S1), we made a blank experiment for phase inversion/precipitation of the pristine NIC solution, which can form stable/quasi-stable colloidal dispersions in the same condition and can later easily become aggregated over time, especially when treated with PBS (Figure S2(1)).

### 2.3. Preparation of BSA-Coated Zein-NIC NPs

Initially, Zein stock solution was produced by dissolving Zein in 70% IPA (Daejung Chemicals, Gyeonggi-do, Korea) with a concentration of 10 mg/mL (Zein stock solution) and NIC was dissolved in IPA with a concentration of 5 mg/mL (NIC stock solution), respectively. Then, 1 mL of dissolved Zein/IPA (containing 10 mg Zein) solution was treated with 200 µL of NIC /IPA (containing 1.25 mg NIC), which was then gently mixed using a vortex mixer (Scientific Industries Inc, Bohemia, NY, USA) for about 1 min. This mixture was directly added into 8.75 mL distilled water containing BSA with a concentration of 20 mg, forming a yellow-colored solution indicative of BSA-Zein-NIC NPs. This solution was further evaporated using a rotary evaporator (Heidolph Instruments Gm bH & Co. KG, Schwabach, Germany) for 10–15 min and then freeze-dried at −53 °C using a freeze drier machine (ILSHIN BIOBASE, Gyeonggi-do, Korea) for 2 days in order to obtain the powder form.

### 2.4. Drug Content Analysis and InVitro Drug Release Studies

In order to understand the NIC drug contents in the formulation, a calibration curve in ethanol was made for NIC, as shown in Figure S3. Thereafter, 3 mg each of Zein-NIC NPs and BSA-Zein-NIC NPs were dissolved in ethanol, which was sonicated for about 30 min to completely dissolve the NIC from the drug-loaded NPs. Thereafter, the solution was syringe-filtered using a 0.22 µm PVDF membrane to get rid of the debris. These were then measured using UV spectrophotometer in order to obtain the absorbance of NIC at 333 nm.

For the *in vitro* drug release, a drug dissolution apparatus was used, set at 37 °C with the RPM maintained at 50 for each sample. The NIC powder, NIC NPs, Zein-NIC NPs, and BSA-Zein NIC NPs with and without 1% serum were tested for the drug release in pH 7.4 buffer (PBS tablets (Gibco^TM^, Thermo Fisher Scientific, Paisley, UK)) were used to make the buffer solution) in order to mimic the normal blood pH. At predetermined time intervals, the samples were withdrawn and syringe-filtered immediately using a 0.22 µm PVDF membrane, and then the absorbance was measured at 333 nm using a UV spectrophotometer.

### 2.5. Colloidal Stability

Colloidal stability was performed in water and phosphate buffered saline for Zein-NIC and BSA-Zein-NIC NPs. For this, freeze-dried powders were used. For each sample, 20 mg was dispersed in 10 mL of water and PBS and syringe-filtered using a PVDF membrane (0.22 µm size). The solutions were analyzed for DLS and Zeta for several days.

### 2.6. Characterizations

DLS and Zeta potential analyses were done using an Otsuka Electronics DLS/Zeta EL-SZ-2000 (Otsuka Electronics, Otsuka, Japan) using disposable square cuvettes in both water and phosphate buffered saline. The FT-IR spectral studies were done with a JASCO FT-IR-6100 spectrometer (JASCO, Tokyo, Japan) using the standard KBr disk method in transmission mode (spectral range 4000–400 cm^−1^, resolution 1 cm^−1^, 40 scans per spectrum). UV-visible spectroscopy data were obtained using a JASCO-V630 Spectrophotometer (JASCO, Easton, MA, USA) and the freeze-drying was carried out with ILSHIN BIOBASE (Gyeonggi-do, Korea) at −53 °C. The XRD patterns for all the samples were obtained with a Bruker D2 Phase Diffractometer (Bruker, Karlsruhe, Germany) equipped with Cu Kα radiation (λ = 1.5418 Å). All the data were recorded at a tube voltage and current of 30 kV and 10 mA, respectively. The field emission scanning electron microscopic analysis for various samples was done using a Sigma 300 field-emission scanning electron microscope (Carl Zeiss, Oberkochen, Germany). The NMR analysis was measured with a Bruker/Magnet System 500′54 Ascend at Dankook University, Cheonan, Korea. DMSO-D6 solvent (Duetero.de, Sejong-si, Korea) was used as the solvent for all the NMR experiments. The *in-vitro* drug release studies were done with a temperature-controlled dissolution apparatus (DST-810 labfine INC., Seoul, Korea). 

### 2.7. Statistical Analysis

The statistical significances of the differences were evaluated with a two-tailed Student’s *t*-test. A *p*-value < 0.05 was considered as statistically significant.

## 3. Results and Discussion

### 3.1. Synthesis and Characterization of Injectable Formulation

#### 3.1.1. XRD, FT-IR, and NMR Analysis

Powder XRD analysis was undertaken in order to understand how NIC encapsulation could affect the overall crystallinity of the present injectable nanoformulation. As expected, both Zein-NIC NPs and BSA-Zein-NIC NPs were found to be amorphously different under X-ray from the intact NIC and its recrystallized forms (see Figure S4). In fact, both characteristic NIC peaks at 2θ = 25.6 and 26.7 degrees totally vanished, indicating that NIC molecules were eventually encapsulated well within the NPs rather than just adsorbed physically.

**Figure 1:**
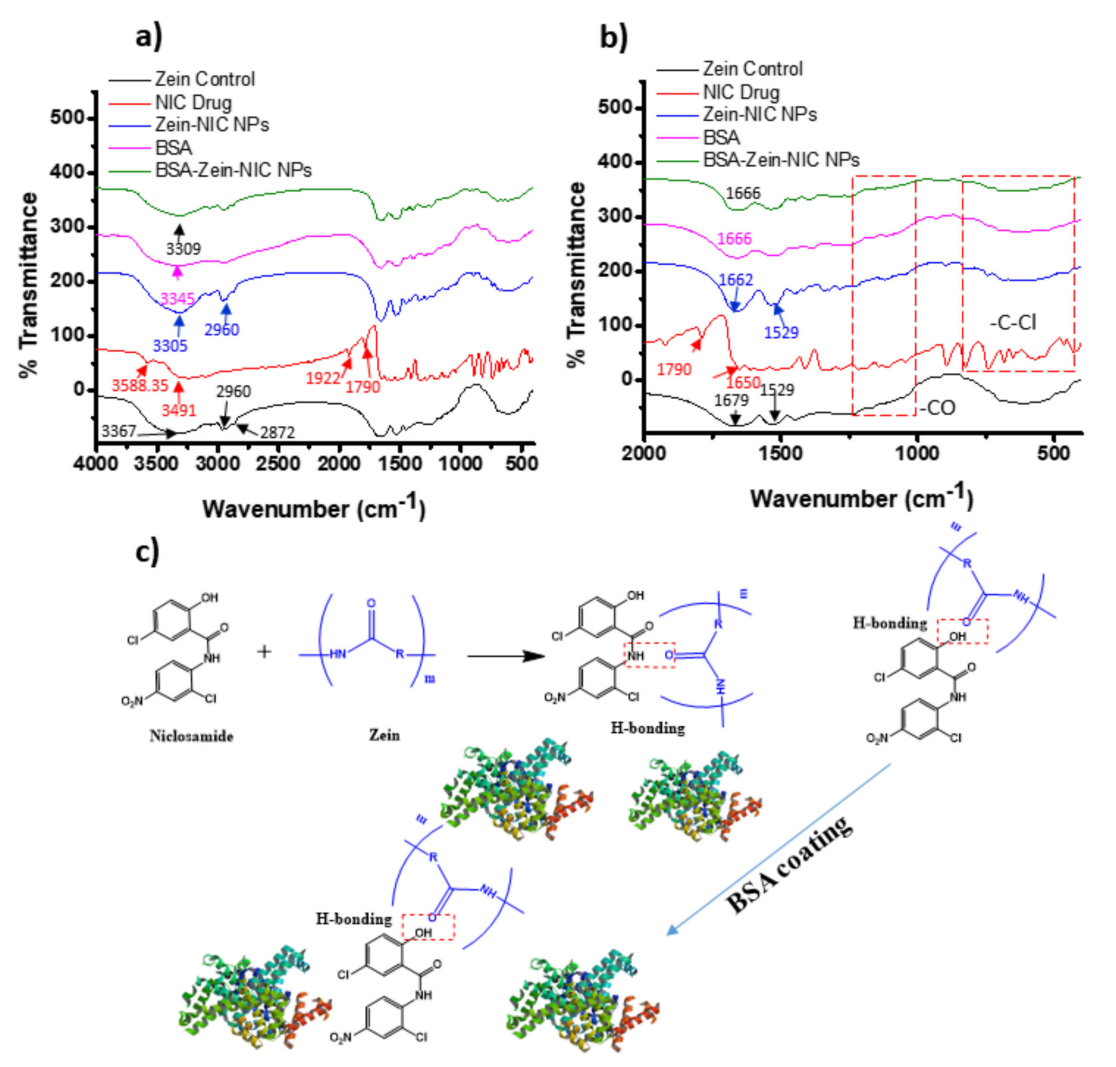
FT-IR analysis for Zein, NIC, Zein-NIC NPs, BSA, and BSA-Zein-NIC NPs: (**a**) full spectrum (4000 to 400 cm^−1^) and (**b**) carbonyl region from 500 to 200 cm^−1^; (**c**) possible interaction between BSA, Zein, and NIC in the final BSA-Zein-NIC NPs.

Further, FT-IR analysis was also done to understand the bonding interaction within the formulations, such as Zein-NIC and BSA-Zein-NIC NPs. One thing to underline here is that the major characteristic peak of NIC at 3588.35 cm^−1^, corresponding to the -NH_2_ stretching, totally vanished in all the developed formulations, likely due to the hydrogen bonding interaction with the peptidic/amide CONH bonds from the Zein macromolecule. The FT-IR spectra in the carbonyl region were also analyzed in order to find if there was any specific shifting in those regions (Figure 1b). The full FT-IR spectra were obtained in order to understand the -CONH, -NH_2_, -OH, and -CH vibrational peaks, specifically from the 2000 to 4000 cm^−1^ region. In general, phenolic hydroxyl groups (Ph-OH) were found to have stretching frequencies of 3700–3584 cm^−1^. In the final BSA-Zein-NIC product, the -OH and -NH peaks seemed to be merged into one broad peak (at 3309 cm^−1^), indicating a combined peak of the hydroxyl group (-OH), which in turn indicated the possibility of intermolecular hydrogen bonding between the major components of the NPs. Since the carbonyl bands generally ranged from 1160 cm^−1^ to 1000 cm^−1^ the bands near 1079 cm^−1^ could be attributed to νCO due to the C-OH ring both in the Zein-NIC NPs and the BSA-Zein-NIC ones. 

The specific bands for NIC with the C-Cl bond were found to be in between 800 and 400 cm^−1^, which were rather weak or vanished after loading on Zein and further coating with BSA. (Figure 1a,b). Furthermore, the carbonyl amide (-CONH) stretching of NIC at around 1650 cm^−1^ was shifted to 1662 cm^−1^, indicating a molecular interaction between -CONH of the NIC ring and the carbonyl groups in Zein molecules [41]. 

Based on the results from the FT-IR, the molecular interactions within the BSA-Zein-NIC NPs are given in Figure 1c, showing the formation of molecular assemblies due to the hydrogen bonding between BSA and Zein-NIC NPs.

To cross-confirm the hydrogen bonding interactions within the BSA-Zein-NIC NPs, a proton NMR analysis was done using DMSO-D6 solvent. The major observations we found in the NMR spectra were displayed in Figure S5. The characteristic peaks for the Zein molecules were found at 6.64 and 7.07 ppm [42], which were slightly shifted downfield after NIC molecule immobilization and BSA coating. Similarly, the aromatic ring protons in NIC were shifted upfield, confirming the involvement of hydrogen bonding interactions.

### 3.2. Particle Size, Zeta, and Surface Morphology Analyses

The particle size of the drug delivery carrier is an important parameter for developing anti-viral medicines, as the formulated particles have to be permeated into the virally infected cells without causing any adverse effects on the neighboring healthy cells. In order to clearly understand the size analysis, we performed DLS analysis for both the Zein-NIC NPs and the BSA-Zein-NIC ones. One thing to note here is that both were prepared with particle sizes ~ 200 nm; the particle size of the Zein-NIC hybrid was optimized with 173 ± 4.5 nm, whereas that of BSA-Zein-NIC was determined to be 207 ± 3.6 nm. A slight increase in particle size for the latter was likely due to the proper coating of BSA to form BSA-Zein-NIC NPs. On the other hand, the control NIC NPs were found to be 149.5 ± 0.97 nm (Figure S6a). The smaller size, of around 200 nm, is highly advantageous for the BSA-Zein-NIC NPs, as these nanohybrid drugs can easily enter into the infected cells and eventually induce anti-viral action. In addition, the damaged endothelial glycocalyx [30,31,32] in COVID-19 patients might further enhance a kind of selective uptake of BSA-coated Zein-NIC NPs [29,30,31,43].

**Figure 2:**
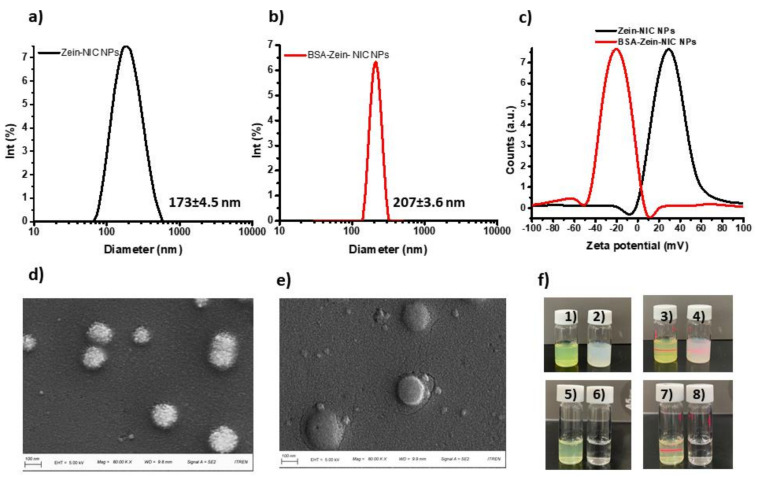
Particle size analysis by DLS and FE-SEM: (**a**) Zein-NIC NPs; (**b**) BSA-Zein-NIC NPs; (**c**) Zeta potential for Zein-NIC NPs and BSA-Zein-NIC NPs in water. FESEM analysis: (**d**) Zein-NIC NPs; (**e**) BSA-Zein-NIC NPs; (**f**) colloidal stability observed for Zein-NIC NPs (2,4,6, and 8) and BSA-Zein-NIC NPs (1,3,5, and 7) before the freeze-drying process (1–4) and after dissolving freeze-dried powder samples in water (5–8). The laser exposure indicated the Tyndall effects shown by stable nano-suspensions.

According to the surface charge analysis undertaken by performing Zeta potential measurements, the surface charge for Zein-NIC NPs was determined to be +29.94 ± 1.74 mV, which did, however, change to negative after forming the BSA-Zein-NIC NPs (−20.00 ± 1.39 mV), as shown in Figure 2c. The control NIC NPs were found to have a Zeta potential value of −15.7 ± 2.24 mV (Figure S6b) indicating an overall negative surface charge. The negative surface charge for NIC was somehow found to be neutralized after assembling with Zein, leaving the outer surface of the Zein-NIC NPs with a positive surface charge. This was again evidence that NIC molecules were well-encapsulated within the Zein core. In addition, it is well-known that BSA molecules have an overall negative surface charge [44], and this could be retained after coating on the Zein-NIC NPs.

Both types of NPs showed a spherical morphology (Figure 2d,e), which was in contrast to the needle/rod-like structure of the NIC NPs alone, as shown in Figure S6c. In fact, the rough surface for the Zein-NIC NPs was converted into a smooth one for BSA-Zein-NIC NPs, confirming the proper physical coating of BSA on the Zein-NIC NPs (Figure 2e).

Further, we observed the colloidal stability for various NPs before and after freeze drying. Interestingly, all the NPs showed good colloidal stability in aqueous solution, which was further improved after coating with the BSA molecules. Compared to NIC NPs and Zein-NIC ones, the BSA-Zein-NIC NPs exhibited significantly lower PDI values, indicating their excellent colloidal stability, which is thought to be very critical for an injectable formulation (Figure 2f, Table 1). In addition, the as-made BSA-Zein-NIC NPs showed good colloidal stability, with a Tindall effect even after 3 weeks, as shown in Figure S1, confirming their suitability as an injectable NIC formulation.

Additionally, we checked the stability in PBS, as shown in Figure S2 and Table S1, which showed that the BSA-Zein-NIC NPs retained stable particle sizes compared to the Zein-NIC NPs. This might have been due to the steric stabilization provided by the BSA coating. Moreover, albumin can act as an active targeting agent as well-documented [45,46,47], therefore the drug-loaded nanoparticles with albumin can selectively target the affected tissue and cells.

### 3.3. In Vitro Drug Release Studies

Prior to drug release studies, the drug contents in the Zein-NIC and BSA-Zein-NIC NPs were analyzed, as shown in the Table 2. 

The NIC content in the Zein-NIC nanohybrid was determined to be ~10.08 ± 0.95%, whereas it decreased down to 3.31 ± 0.041% after coating with BSA, indicating that the BSA molecules were well-coated on the external surface of the Zein-NIC NPs to form BSA-Zein-NIC NPs. Accordingly, the actual amount of NIC in 1 mg of Zein-NIC NPs was determined to be ~0.0986 ± 0.003 mg (*w*/*w*), whereas 1 mg of BSA-Zein-NIC NPs was comprised of ~0.0326 ± 0.0009 mg NIC. 

**Figure 3:**
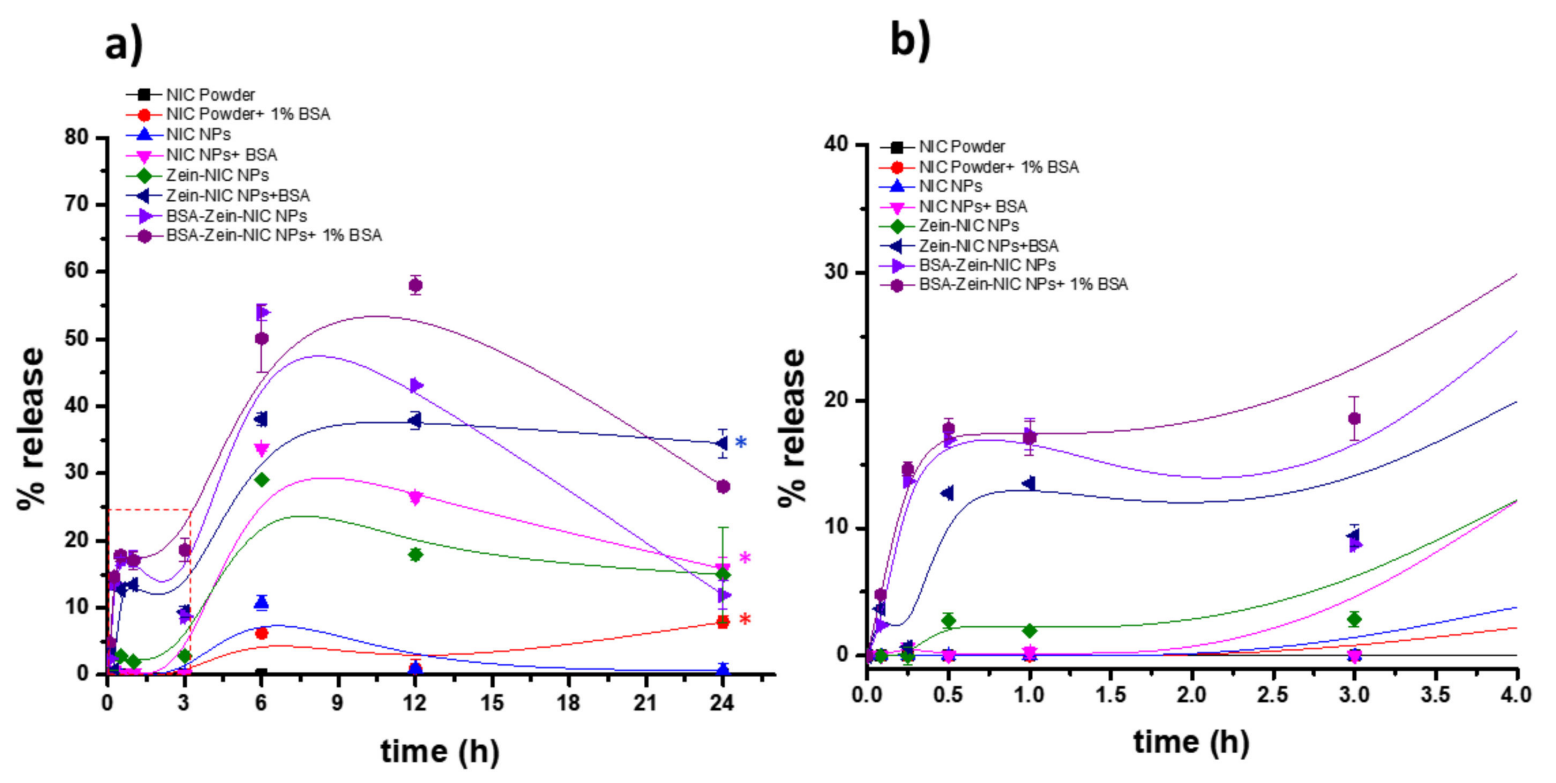
*In vitro* NIC release for NIC powder, NIC NPs, Zein-NIC NPs, and BSA-Zein-NIC NPs under serum-free and serum-treated conditions in pH 7.4 buffer solution: (**a**) 0 to 24 h and (**b**) initial 3 h results (*n* = 3, * represents *p* < 0.05).

The *in vitro* drug release studies at pH 7.4 were undertaken with various samples, namely NIC (powder), NIC-NPs, Zein-NIC NPs, and BSA-Zein-NIC NPs with and without 1% serum containing dissolution media. It became very clear that the BSA-Zein-NIC NPs showed a comparatively high NIC release rate compared to that of the other samples, as mentioned above. A maximum of 60% NIC release was observed with the serum-containing buffer (pH 7.4), which was almost similar in terms of the NIC release to the serum-untreated sample of the BSA-Zein-NIC NPs (Figure 3). As expected, NIC in the powder form, as well the NIC NPs, showed a significantly low release, especially in the case of the NIC powder, due to the fact that NIC is hydrophobic in nature and, therefore, cannot be dissolved well in aqueous solution. On the other hand, the addition of serum resulted in a slight increase in the dissolution. Similarly, the NIC releases from Zein-NIC NPs and NIC NPs were also significantly improved after serum treatment. However, the NIC release from BSA-Zein-NIC NPs with and without serum treatment showed no difference, which was logically to be expected, mainly because there was already a kind of serum coating on the BSA-Zein-NIC NPs, so the extra serum coating would not make any difference. It should also be noted that the major component of commercially available, lab-based, cultural fetal bovine serum is albumin.

After 24 h, the NIC release was found to be saturated and then reduced. The major mechanism that meant that NIC could be released out of BSA-Zein-NIC NPs dramatically better than from the other samples can be explained based on two factors: the first is the BSA coating, which can not only improve stability, but also enhances and controls the NIC release effectively. The BSA molecules became negatively charged in an aqueous solution, meaning that they became swellable in an aqueous solution. Secondly, the Zein shell, which can undergo swelling in an aqueous medium, also improves the NIC release from the nanohybrid matrix.

## 4. Clinical Perspectives

Despite the various approaches to developing new anti-viral drugs for combating COVID-19, there have been only a few studies related to injectable NIC formulations, which has prompted scientists to come up with novel therapeutic NIC formulations. For example, NIC was made into a PEGylated injectable formulation by Ma et al. (2019), though there was no information provided regarding the actual particle size and morphology of the developed injectable NIC formulation [48]. Recently, our group reported new oral formulations for NIC, showing excellent PK results compared with many other related studies [26]. Here, instead of focusing on oral formulations, our main goal was to develop an injectable formulation for NIC, which should have the selectivity as well as the sustainability for better therapeutic outcomes. These were the main reasons why we specifically chose Zein and BSA as the core and shell biomaterials for repurposing the NIC drug towards treatment of COVID-19. We came up with the idea that a hydrophobic drug like NIC could be protected inside the Zein and then released outside it in a sustained manner, while the outer coating of BSA would help in carrying the NIC molecules selectively to the virus-infected cells.

In fact, the Zein-based nanomaterials have been found to possess excellent swelling properties in an aqueous solution, which is an added advantage along with their high biocompatibility [49]. For example, Berardi et al. reported the swelling behavior of Zein, finding that it could be enhanced with the addition of co-excipients [50]. Taking advantage of this, we hypothesized that once the NIC molecules are encapsulated inside the Zein and injected after the BSA coating, they would work selectively, getting taken up by the damaged endothelial glycocalyx present in most elderly COVID-19 patients. With regard to future research perspectives, we are thinking about precision medicine with NIC based on patients’ own serum protein instead of commercially available BSA, which would further improve the therapeutic outcome. 

## 5. Conclusions

In conclusion, we systematically developed highly stable BSA-coated Zein-NIC NPs as an injectable nanomedicine, which could be useful in COVID-19 therapy. The as-made BSA-Zein-NPs showed an optimum particle size of ~200 nm which was stable under the serum condition. Further, we confirmed the interaction of BSA, Zein, and NIC within the nano-assembly using FT-IR, XRD, and NMR. The *in vitro* release studies indicated that only the BSA-Zein-NIC NPs showed the sustained NIC release, and it was significantly higher than the intact NIC NPs and the uncoated Zein-NIC NPs at pH 7.4 in an aqueous solution, indicating that BSA-Zein-NIC NPs could serve as a potential candidate for injectable nanomedicine. Since the albumin-coated NPs could be targeted via a damaged endothelial glycocalyx in COVID-19 patients, we think that the described injectable BSA-Zein-NIC formulation will be very advantageous. Further studies are necessary to understand the pharmacokinetics and the anti-viral effect of this formulation on SARS-CoV-2 infected cells/animals, which will be undertaken in the very near future.

## Figures and Tables

**Table 1:** Colloidal stability in terms of PDI values of various NPs in aqueous solution, namely NIC, Zein-NIC, and BSA-Zein-NIC. *n* = 3, ** *p*-value < 0.005.

Samples	PDI (a.u.)
NIC NPs	0.385 ± 0.026
Zein-NIC NPs	0.314 ± 0.013
BSA-Zein-NIC NPs	0.032 ± 0.005 **

**Table 2:** Drug content analysis of Zein-NIC and BSA-Zein-NIC NPs (*n* = 3).

Samples	Drug Content (%)
Zein-NIC NPs	10.08 ± 0.95
BSA-Zein-NIC NPs	3.31 ± 0.041

## Data Availability

The data presented in this research study are available in this article.

## Supplementary Materials

The following are available online at https://www.mdpi.com/article/10.3390/ma14143792/s1, Figure S1. *In vitro* colloidal stability of various samples in water at RT on day 1 (a), day 7 (b), and day 21 (c) for NIC NPs (1), Zein-NIC NPs (2), and BSA-Zein-NIC NPs (3), where, even after 3 weeks at RT, the Tindall effect was still shown only by the BSA-Zein-NIC NPs; Figure S2. *In vitro* stability in PBS for (1) NIC NPs, (2) Zein-NIC NPs, and (3) BSA-Zein-NIC NPs on days 1, 2, and 3 respectively. The Tindall effect was significantly higher in the BSA-Zein-NIC NPs due to the steric stabilization provided by the BSA coating on the Zein-NIC NPs; Figure S3. NIC calibration in ethanol; Figure S4. XRD analysis: (a) control BSA; (b) NIC; (c) Zein; (d) recrystallized NIC; (e) Zein-NIC NPs; and (f) BSA-Zein-NIC NPs; Figure S5. Proton NMR analysis for various samples: (a) intact NIC; (b) Zein; (c) Zein-NIC NPs; (d) BSA; and (e) BSA-Zein-NIC NPs; Figure S6. (a) DLS analysis, (b) Zeta potential, and (c) FE-SEM for NIC NPs prepared in an ethanol/water mixture followed by probe sonication at 40% amplitude for 60 s; Table S1. Stability analysis for Zein-NIC and BSA-Zein-NIC NPs in phosphate buffered saline.
