# Supplementary material for: Bovine Serum Albumin-Coated Niclosamide-Zein Nanoparticles as Potential Injectable Medicine against COVID-19
Source: Materials (Basel). 2021 Jul 7;14(14):3792. doi: 10.3390/ma14143792 (PMC8307271; doi:10.3390/ma14143792)
Supplement: Supplementary file 1 [file materials-14-03792-s001.zip › materials-1269403-supplementary.pdf]

materials

Supporting Information

Bovine Serum Albumin-Coated Niclosamide-Zein Nanoparticles as Potential Injectable Medicine against COVID-19

Sanoj Rejinold N [1], Goeun Choi [1,2,3], Huiyan Piao [1] and Jin-Ho Choy [1,4,5,*]

1 Intelligent Nanohybrid Materials Laboratory (INML), Institute of Tissue Regeneration Engineering (ITREN), Dankook University, Cheonan 31116, Korea; sanojrejinold@dankook.ac.kr (S.R.N.); goeun.choi@dankook.ac.kr (G.C.); 12192032@dankook.ac.kr (H.P.)
2 College of Science and Technology, Dankook University, Cheonan 31116, Korea
3 Department of Nanobiomedical Science and BK21 PLUS NBM Global Research Center for Regenerative Medicine, Dankook University, Cheonan 31116, Korea
4 Department of Pre-medical Course, College of Medicine, Dankook University, Cheonan 31116, Korea
5 Tokyo Tech World Research Hub Initiative (WRHI), Institute of Innovative Research, Tokyo Institute of Technology, Yokohama 226-8503, Japan
* Correspondence: jhchoy@dankook.ac.kr

Citation: Rejinold, N.S.; Choi, G.; Piao, H.; Choy, J.-H. Bovine Serum Albumin-Coated Niclosamide-Zein Nanoparticles as Potential Injectable Medicine Against COVID-19. *Materials* **2021**, *14*, 3792. https://doi.org/10.3390/ma14143792

Academic Editor: Marilena Vlachou

Received: 5 June 2021
Accepted: 1 July 2021
Published: 7 July 2021

Publisher's Note: MDPI stays neutral with regard to jurisdictional claims in published maps and institutional affiliations.

Copyright: © 2021 by the authors. Submitted for possible open access publication under the terms and conditions of the Creative Commons Attribution (CC BY) license (http://creativecommons.org/licenses/by/4.0/).

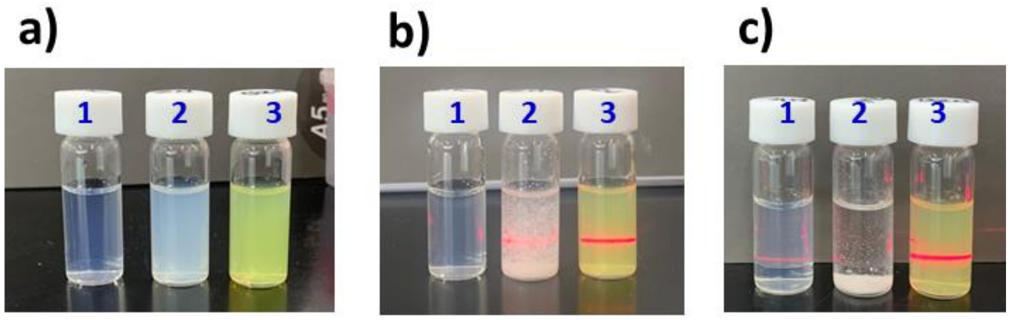

Figure S1. In-vitro colloidal stability of various samples in water at RT on day 1 (a), day 7 and c) day 21 for NIC NPs (1), Zein-NIC NPs (2) and BSA-Zein-NIC NPs (3), where, even after a 3 weeks at RT, the Tindall effect was still shown only by the BSA-Zein-NIC NPs.

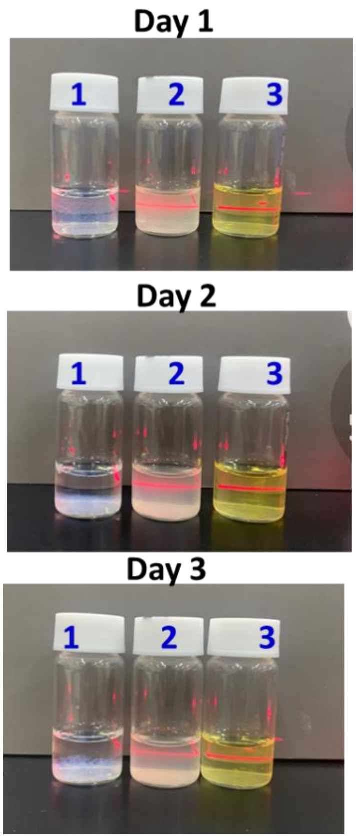

Figure S2. *In-vitro* stability in PBS for (1) NIC NPs, (2) Zein-NIC NPs and (3) BSA-Zein-NIC NPs on day 1, 2 and 3 respectively. The Tindall effect was significantly higher in the BSA-Zein-NIC NPs due to the steric stabilization provided by the BSA coating on Zein-NIC NPs.

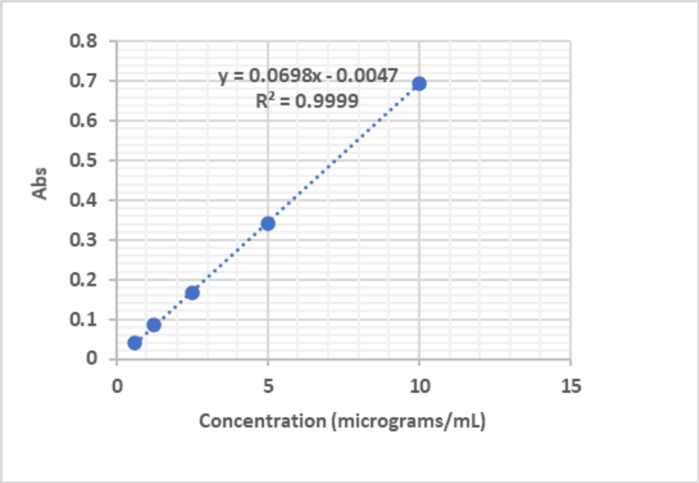

Figure S3. NIC calibration in ethanol.

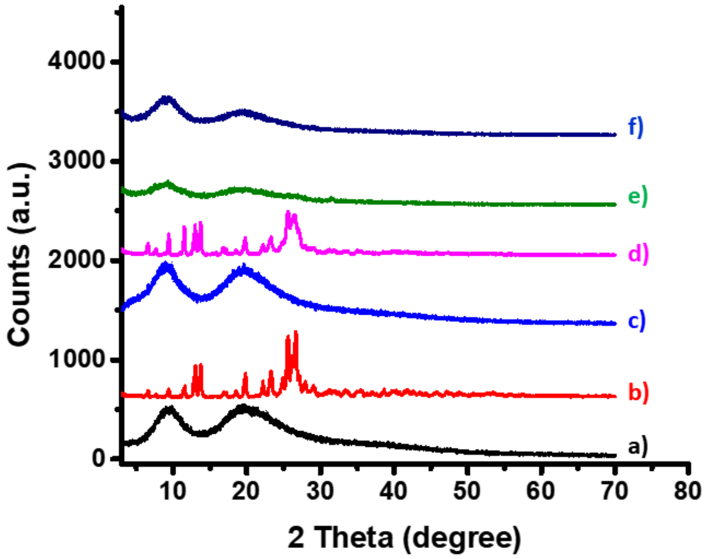

Figure S4. XRD analysis: (a) control BSA; (b) NIC ;(c) Zein; (d) recrystallized NIC; (e) Zein-NIC NPs and (f) BSA-Zein-NIC NPs.

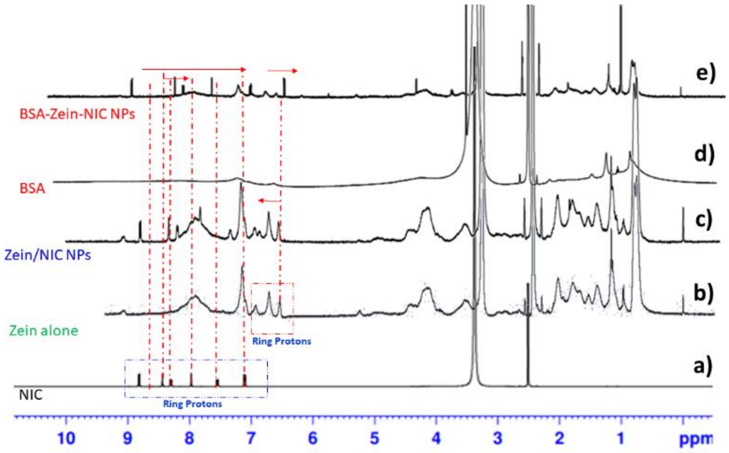

Figure S5. Proton NMR analysis for various samples such as a) intact NIC; b) Zein ; c) Zein-NIC NPs; d) BSA; and e) BSA-Zein-NIC NPs.

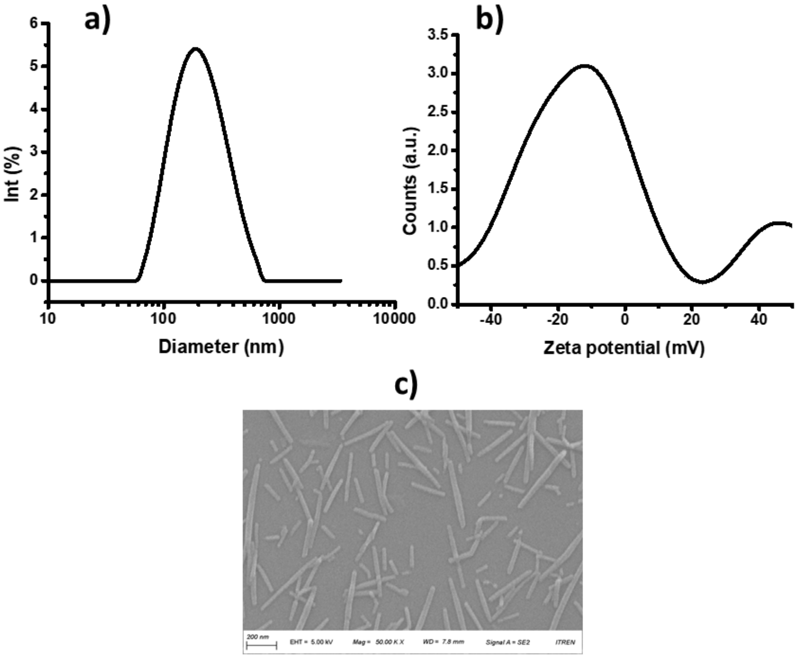

Figure S6. (a) DLS analysis, (b) Zeta potential, and c) FE-SEM for NIC NPs prepared in ethanol/water mixture followed by probe sonication at 40% amplitude for 60 seconds.

Table S1. Stability analysis for Zein-NIC and BSA-Zein-NIC NPs in phosphate buffered saline.

Sample	Day	Size in diameter (nm)	PDI (a.u.)
Zein-NIC-NPs	1	813.00 ± 115	0.4315 ± 0.062
Zein-NIC NPs	2	667.00 ± 1.48	0.0354 ± 0.027
Zein-NIC NPs	3	512.45 ± 4.87	0.0301 ± 0.004
BSA-Zein-NIC-NPs	1	261.60 ± 6.50	0.3385 ± 0.012
BSA-Zein-NIC NPs	2	223.75 ± 1.34	0.3905 ± 0.013
BSA-Zein-NIC NPs	3	237.20 ± 5.51	0.3745 ± 0.009
